# ICT communication with family/friends and its associations with mental health and insomnia in COVID-19 patients during hospital quarantine: a cross-sectional study in Shenzhen, China

**DOI:** 10.1186/s12888-025-07079-6

**Published:** 2025-07-01

**Authors:** Tie Cheng Liu, Zi Yuan Yu, Shi Wen Lin, Rui Heng Nong, Yi Na Lu, Chen Rui Wang, Jia Jing Zeng, Peng Fei Li, Li Jun Zhong, Shi Tong Xu, Wei Jie Gong

**Affiliations:** 1 South China Hospital, Medical School, Shenzhen University, Shenzhen, Guangdong China; 2 School of Public Health, Medical School, Shenzhen University, Shenzhen, Guangdong China; 3 School of Nursing, Medical School, Shenzhen University, Shenzhen, Guangdong China; 4 Department of Family Medicine, Medical School, Shenzhen University, Shenzhen, Guangdong China

**Keywords:** COVID-19 patient, Hospital quarantine, Information and communication technology, Insomnia, Mental health

## Abstract

**Background:**

Information and communication technology (ICT) use can help isolated people buffer mental health burdens, but little evidence was in patients during hospital quarantine. We aimed to examine how COVID-19 patients used ICT to communicate with family/friends and its associations with their mental health and insomnia during hospital quarantine.

**Methods:**

This cross-sectional study involved quarantined COVID-19 Chinese patients in Shenzhen during 10 March-28 April, 2022 who reported their ICT communication with family/friends, including text messages, picture messages, short videos, voice calls, video calls, and e-mails. The associations of ICT communication with sociodemographic characteristics, mental health, and insomnia were examined using adjusted prevalence ratios (aPR). We used lower cutoffs to identify mild psychological symptoms and insomnia for early prevention and higher cutoffs of their clinical diagnoses as sensitivity analyses.

**Results:**

Among the 217 respondents (female 48.9%, 25–54 years 63.1%), the most common ICT use was text messages (72.8%), voice calls (57.1%), and video calls (53.5%). More females used picture messages (aPR 2.06), fewer older patients used text (0.68) and picture messages (0.42), and more patients with tertiary/above education levels used text messages (1.23), video calls (1.47), and picture messages (1.82) (all *P* ≤ 0.05). Using any or more types of ICT was associated with less depression, anxiety, post-traumatic, somatization symptoms, and insomnia (0.31–0.55 and 0.67–0.85, respectively, all *P* ≤ 0.001), especially using video calls (0.36–0.76, *P* ≤ 0.05). Similar results were found in the sensitivity analyses.

**Conclusions:**

Hospital-quarantined COVID-19 patients using any or more types of ICT to communicate with family /friends tended to have fewer mental health problems and insomnia, especially using video calls.

**Supplementary Information:**

The online version contains supplementary material available at 10.1186/s12888-025-07079-6.

## Background

The COVID-19 pandemic has caused a variety of physical and mental health problems worldwide [1]. Measures to control its spread, including lockdowns, social distancing, and hospital quarantine, brought multiple inconveniences to people’s daily lives, increased social isolation, and added additional sources of stressors to mental health [2]. Isolation hinders people from exchanging information and engaging in regular social interactions and largely influences people’s daily lives. Prolonged confinement in a limited space can lead to a range of physical and mental health issues, such as loneliness, anxiety, depression, and even insomnia [3, 4]. Despite the hard lessons and significant losses experienced during the quarantined periods, the COVID-19 pandemic was still far from over in 2023 [5]. The post-pandemic world continues to face threats from various respiratory infectious diseases, including COVID-19 variants and H1N1 influenza [6]. Social restrictions may need to be implemented again in the future if necessary. Therefore, evidence on effective ways to reduce social isolation and improve mental health should be highly valued.

With the penetration of the Internet and the high prevalence of smartphone ownership, information and communication technology (ICT) use has become an essential part of daily life. ICT tools include smartphones, wearable mobile devices, tablet computers [7], various communication software, mobile networks, and other communication functions. ICT tools enable individuals to maintain social connections and exchange information with family and friends efficiently, irrespective of geographical barriers [8]. Therefore, ICT may help alleviate mental problems caused by isolation [9–11]. Previous studies have shown that proper ICT use can relieve stress, loneliness, anxiety, and insomnia [10, 11], especially during the COVID-19 pandemic [12]. However, little is known about how patients in hospital quarantine used ICT tools.

As one of China’s most developed cities, Shenzhen has taken active response measures to protect people’s health during the pandemic, such as home quarantine, social isolation, and lockdowns. However, long-time quarantine may also bring negative impacts on people’s mental health [13], and even cause post-traumatic stress disorder (PTSD) symptoms and somatic symptoms [14, 15]. Using ICT to communicate with family/friends may help alleviate such adverse effects [16], but evidence on quarantined patients is scarce. Therefore, this study aims to examine what ICT tools were used to communicate with family/friends during COVID-19 patients’ hospital quarantine and their associations with mental health and insomnia. We propose the following hypotheses: (1) hospital-quarantined COVID-19 patients who used ICT to communicate with family/friends would experience lower levels of depression, anxiety, PTSD, somatization symptoms, and insomnia, (2) different types of ICT communication might have varying effects on mental health and insomnia, with video calls being expected to be more effective in reducing psychological distress and improving sleep quality.

## Methods

### Study design

This cross-sectional study was conducted in Shenzhen, a prominent economic center in China that holds significant importance as a critical gateway for international travel and trade. From 10 January 2020 (the first COVID-19 case in Shenzhen) to October 2022, before the first wave of the COVID-19 pandemic (November 2022-January 2023) in Shenzhen, all individuals who tested positive for COVID-19 were first admitted and treated in Shenzhen Third People’s Hospital, the largest local infectious disease hospital in Shenzhen. Since 26 February 2022, once their tests turned negative for COVID-19 and their symptoms became mild, they were then transferred to the South China Hospital to undergo the final 14-day hospital quarantine. On the first day of this final quarantine during 10 March − 28 April 2022, they were invited by short e-messages to voluntarily answer the self-administrated online questionnaire using the online survey tool Questionnaire Star (http://www.wjx.cn). All participants gave written informed consent before answering the questionnaire and agreed with the use of their data in academic studies and publications. Ethics approval [No: 20220310001-A] was granted by the Ethics Committee of the South China Hospital of Shenzhen University. All data was anonymous and no identifiable information was collected. Whether they completed the questionnaire or not had no relation to individuals’ treatment and hospital quarantine.

### Participants

Considering the quarantine policy, we used the convenience sampling method in this study. Only patients who met the following inclusion criteria were invited to voluntarily answer the e-questionnaire via short e-message invitations: (1) their tests for COVID-19 had turned negative, and their symptoms had become mild after hospital treatment; (2) they were currently quarantined at the South China Hospital; (3) they had mobile communication devices such as mobile phones or tablets. Those who could not independently complete the questionnaire were excluded.

**Fig. 1:**
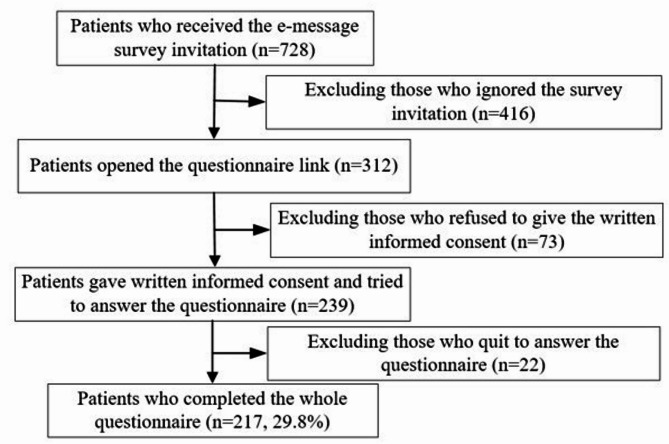
Flowchart of the study sample

As shown in Fig. 1, a total of 728 qualified patients received the e-message invitation, of which 312 (42.9%) opened the questionnaire link, 239 (32.8%) patients gave written informed consent and tried to answer the questionnaire, and finally, 217 (29.8%) voluntarily completed the whole questionnaire. According to the 10 events per variable (10 EPV) rule of thumb for developing a clinical prediction model [17–19], we have a maximum of 10 predictor parameters in the used regression models. Thus, a minimum sample size of 100 would be required. Our final sample of 217 participants therefore provides adequate statistical power for the planned analyses.

## Measurements

### Sociodemographic characteristics

Sociodemographic characteristics were collected, including sex (female and male), age (< 25 years, 25–54 years, and ≥ 55 years), education levels (secondary or below, tertiary or above), marital status (single, married or cohabitated, widowed or divorced), monthly household income (< CNY ¥5000, 5000–9999, and ≥ CNY ¥10000) (USD $1 = CNY ¥7.15), and household registration (local and non-local).

### ICT use

ICT use to communicate with family/friends were asked by the question “Over the past 14 days, what did you usually use to communicate with your family and/or friends?” with multiple choices including no communication, text messages, picture messages, short videos, voice calls, video calls, and emails.

### Depression

Depression was assessed by the Chinese version of the Patient Health Questionnaire (PHQ-9) [20], which has been widely accepted as a valid and reliable instrument for quantifying the severity of depressive symptoms [21]. It consists of 9 items with a 4-point Likert scale. The maximum score is 27, with higher scores indicating more severe depression [21]. The PHQ-9 showed good reliability in this study with a Cronbach’s alpha of 0.91 [22].

### Anxiety

Anxiety was assessed using the Chinese version of the Generalized Anxiety Disorder Scale (GAD-7), which has shown good validity and reliability [22]. It consists of 7 items with a 4-point Likert scale. The maximum score is 21, with higher scores indicating more severe anxiety. In this study, the Cronbach’s alpha of GAD-7 was 0.95 [23].

### Insomnia

Insomnia was assessed using the Chinese version of the Insomnia Severity Index (ISI), which has good validity and reliability [23], and has been previously used to assess psychological well-being in the aftermath of the COVID-19 pandemic [24]. It consists of 7 items with a 4-point Likert scale. The maximum score is 28, with higher scores indicating more severe insomnia [24]. The ISI showed good reliability in this study with a Cronbach’s alpha of 0.94 [25].

### Post-traumatic stress disorder

PTSD was assessed by the Chinese version of PTSD Checklist-Civilian version (PCL-C) [25], which has been demonstrated strong reliability and validity. It consists of 17 items with a 5-point Likert scale. The score range is 17 to 85, with higher scores indicating more severe PTSD [26]. In this study, the Cronbach’s alpha of PCL-C is 0.96 [28].

### Somatization symptoms

Somatization symptoms were assessed by the Somatic Symptom Scale-China (SSS-CN) [27], which has shown favorable reliability and factorial validity [27]. It consists of 20-item with a 4-point Likert scale. The score range is 20 to 80, with higher scores indicating more severe somatization symptoms. In this study, the SSS-CN has shown good reliability with a Cronbach’s alpha of 0.94 [28].

### Statistical analysis

The number of ICT use was calculated by the total number of options chosen for “ICT use” except for the option of “no communication”. Continuous and categorical variables were described by mean ± standard deviation (Mean ± SD) and frequency (percentage), respectively. Poisson regression models with robust variance estimators were used to estimate the associations of different ICT use to communicate with family/friends with all binary outcomes using adjusted prevalence ratios (aPRs) and 95% confidence intervals (CIs) [28], adjusting for sex, age, education levels, marital status, monthly household income, and household registration. Patients with SSS-CN scores ≥ 30 were considered as having somatization symptoms [29]. From the view of early prevention using costless measures, we used relatively lower cutoffs to define the mild symptoms of the other binary outcomes, including depression (≥ 5) [29], anxiety (≥ 5) [30], PTSD (≥ 38) [31], and insomnia (≥ 8) [32]. To examine the robustness of the results, we also conducted a sensitive analysis using relatively higher cutoffs to define the clinically significant conditions of these outcomes, including depression (≥ 10) [33], anxiety (≥ 10) [34], PTSD (≥ 44) [35], and insomnia (≥ 11) [36]. All analyses were conducted using STATA version 13.0. A 2-sided α < 0.05 was considered statistically significant.

## Results

Of the 728 patients who received the questionnaire invitation, 217 (29.8%) completed the questionnaire. Table 1 shows that 51.2% of them were male, 63.1% were aged 25–54 years, 58.5% were married or cohabitating, 73.7% had an education level as secondary or below, 30.4% had a monthly household income of < CNY ¥5,000, and 34.1% had ≥ CNY ¥10,000 Yuan. Most respondents (86.6%) were local residents in Shenzhen.

**Table 1:** Sociodemographic characteristics of the study sample, *N* = 217

Sociodemographic characteristics	*n* (%) ^a^
**Sex**	
Female	106 (48.9)
Male	111 (51.2)
**Age**,** years**	
< 25	44 (20.3)
25–54	137 (63.1)
≥ 55	36 (16.6)
**Education levels**	
Secondary or below	160 (73.7)
Tertiary or above	57 (26.3)
**Monthly household income**,** CNY**^**b**^	
< 5000	77 (35.5)
5000–9999	66 (30.4)
≥ 10,000	74 (34.1)
**Marital status**	
Single	75 (34.6)
Married or cohabitated	127 (58.5)
Widowed or divorced	15 (6.9)
**Household registration**	
Local	188 (86.6)
Non-local	29 (13.7)

^a^Total percentages may not be 100.0% after rounding

^b^USD $1 = CNY ¥7.18

Table 2 shows that the most commonly used ICT tool to communicate with family/friends was text messages (72.8%), followed by voice calls (57.1%), video calls (53.5%), picture messages (29.5%), short videos (22.6%), and emails (1.4%). On average, respondents used 2.4 ± 1.46 types of ICT tools to communicate with family/friends.

**Table 2:** Percentages of using different ICT tools to communicate with family/friends by sociodemographic characteristics, n (%), *N*= 217

Sociodemographic characteristics	Any^a^	Text messages	Voice calls	Video calls	Picture messages	Short videos	Emails
	209 (96.30)	158 (72.80)	124 (57.10)	116 (53.50)	64 (29.50)	49 (22.60)	3 (1.40)
**Sex**							
Male	108 (49.76)	77 (35.48)	65 (29.93)	57 (26.29)	22 (10.14)	19 (8.76)	2 (0.93)
Female	101 (46.54)	81 (37.32)	59 (27.17)	59 (27.21)	42 (19.36)	30 (13.84)	1 (0.47)
**Age**,** years**							
< 25	43 (19.81)	34 (15.67)	31 (14.28)	26 (11.99)	15 (6.91)	8 (3.69)	0 (0)
25–54	133 (61.28)	105 (48.38)	74 (34.08)	75 (34.59)	42 (19.36)	38 (17.53)	0 (0)
≥ 55	33 (15.21)	19 (8.75)	19 (8.75)	15 (6.92)	7 (3.23)	3 (1.38)	3 (1.40)
**Education levels**							
Secondary or below	153 (70.50)	109 (50.22)	84 (38.68)	77 (35.51)	39 (17.98)	35 (16.14)	1 (0.47)
Tertiary or above	56 (25.80)	49 (22.58)	40 (18.42)	39 (17.99)	25 (11.52)	14 (6.46)	2 (0.93)
**Monthly household income**,** CNY**^**b**^							
< 5000	72 (33.18)	54 (24.88)	39 (17.96)	35 (16.14)	22 (10.14)	10 (4.61)	0 (0)
5000–9999	65 (29.95)	48 (22.12)	38 (17.50)	38 (17.53)	19 (8.76)	18 (8.30)	1 (0.47)
≥ 10,000	72 (33.18)	56 (25.80)	47 (21.64)	43 (19.83)	23 (10.60)	21 (9.69)	2 (0.93)
**Marital status**							
Single	72 (33.18)	59 (27.18)	52 (23.95)	42 (19.37)	22 (10.14)	12 (5.53)	0 (0)
Married or cohabitated	123 (56.67)	89 (41.01)	66 (30.39)	72 (33.21)	38 (17.52)	34 (15.68)	3 (1.40)
Widowed or divorced	14 (6.45)	10 (4.61)	6 (2.76)	2 (0.92)	4 (1.84)	3 (1.38)	0 (0)
**Household registration**							
Local	181 (83.40)	136 (62.66)	103 (47.43)	99 (45.60)	49 (22.59)	41 (18.91)	2 (0.93)
Non-local	28 (12.90)	22 (10.14)	21 (9.67)	17 (7.84)	15 (6.91)	8 (3.69)	1 (0.47)

ICT, information and communication technologies. SD, standard deviation. ^a^Any: using any ICT tools to communicate with family/friends. ^b^USD $1 = CNY ¥7.18

More females used picture messages to communicate with family/friends (aPR 2.06, *P* = 0.001) (Table 3). Patients aged ≥ 55 years used less text messages (0.68, *P* for trend = 0.03), picture messages (0.42, *P* for trend = 0.16), and short videos (0.21, *P* for trend = 0.22). Those with tertiary or above education levels used more text messages (1.23, *P* = 0.03), picture messages (1.47, *P* = 0.005), and video calls (1.82 *P* = 0.01). Those with higher monthly household incomes used more short videos (aPRs 2.13–2.69, *P* for trend < 0.05). The associations of marital status and household registration with ICT use showed no statistical significance (all *P* ≥ 0.05).

**Table 3:** Characteristics linked to ICT use for family/friends communication, aPR (95% CI), *N* = 217

Sociodemographic characteristics	Any^a^	Text messages	Voice calls	Video calls	Picture messages	Short videos
**Sex**						
Male	1	1	1	1	1	1
Female	0.98 (0.93, 1.03)	1.12 (0.96, 1.31)	1.03 (0.82, 1.31)	1.17 (0.92, 1.49)	2.06 (1.35, 3.15) ***	1.47 (0.87, 2.49)
**Age**,** years**						
< 25	1	1	1	1	1	1
25–54	0.97 (0.89, 1.05)	0.95 (0.76, 1.17)	0.83 (0.62, 1.12)	0.80 (0.57, 1.12)	0.64 (0.38, 1.08)	0.86 (0.39, 1.89)
≥ 55	0.90 (0.80, 1.01)	0.68 (0.47, 0.96) *	0.85 (0.54, 1.34)	0.64 (0.38, 1.07)	0.42 (0.18, 1.00) *	0.21 (0.05, 0.84) *
**Education levels**						
Secondary or below	1	1	1	1	1	1
Tertiary or above	1.03 (0.95, 1.11)	1.23 (1.02, 1.49) *	1.15 (0.87, 1.51)	1.47 (1.12, 1.93) **	1.82 (1.13, 2.92) *	1.10 (0.57, 2.13)
**Monthly household income**,** CNY**^**b**^						
< 5000	1	1	1	1	1	1
5000–9999	1.05 (0.98, 1.12)	1.02 (0.84, 1.25)	1.08 (0.80, 1.47)	1.11 (0.82, 1.51)	0.86 (0.52, 1.42)	2.13 (1.01, 4.50) *
≥ 10,000	1.04 (0.98, 1.11)	1.06 (0.88, 1.32)	1.23 (0.93, 1.64)	1.19 (0.87, 1.60)	1.02 (0.62, 1.67)	2.39 (1.14, 5.02) *
**Marital status**						
Single	1	1	1	1	1	1
Married or cohabit	1.05 (0.97, 1.14)	1.01 (0.84, 1.21)	0.84 (0.62, 1.13)	1.27 (0.92, 1.77)	1.51 (0.89, 2.55)	2.01 (0.97, 4.17)
Divorce or widow	1.04 (0.89, 1.21)	0.95 (0.64, 1.40)	0.65 (0.34, 1.25)	0.30 (0.08, 1.10)	1.12 (0.45, 2.78)	1.94 (0.63, 5.99)
**Household registration**						
Yes	1	1	1	1	1	1
No	0.95 (0.88, 1.01)	0.97 (0.55, 0.86)	1.20 (0.90, 1.58)	0.94 (0.65, 1.35)	1.55 (0.10, 0.33)	1.27 (0.62, 2.61)

All variables were mutually adjusted. * *P* < 0.05, ** *P* < 0.01, *** *P* < 0.001. ICT, Information and Communication Technologies. aPR, adjusted prevalence ratio. CI, confidence interval. ^a^ Using any types of ICT tools to communicate with family/friends. ^b^USD $1 = CNY ¥7.18

Table 4 shows that using any ICT tools to communicate with family/friends was associated with lower odds of all outcomes, including depression, anxiety, post-traumatic syndrome, somatization symptoms, and insomnia (aPRs 0.31–0.55, all *P* ≤ 0.001), so as using more types of ICT tools (aPRs 0.67–0.85, all *P* ≤ 0.05) and video calls (aPRs 0.36–0.73, all *P* ≤ 0.02). Using voice calls was associated with lower odds of depression (aPR 0.76, *P* = 0.03), anxiety (aPR 0.72, *P* = 0.02), somatization symptoms (aPR 0.69, *P* = 0.028), and insomnia (aPR 0.76, *P* = 0.032). Using picture messages was associated with lower odds of depression (aPR 0.66, *P* = 0.01), anxiety (aPR 0.69, *P* ≤ 0.05) and insomnia (aPR 0.66, *P* = 0.016). Using text messages was only associated with lower odds of anxiety (aPR 0.75, *P* = 0.04). The associations of using short videos and emails with depression, anxiety, PTSD, somatization symptoms, and insomnia showed no statistical significance (all *P* ≥ 0.23).

**Table 4:** Using different ICT tools to communicate with family/friends links to mental health, insomnia, and somatization, (*N* = 206)

Different ICT tools	Depression		Anxiety		Post-traumatic syndrome		Somatization symptoms		Insomnia	
	*n* (%)	aPR (95% CI)	*n* (%)	aPR (95% CI)	*n* (%)	aPR (95% CI)	*n* (%)	aPR (95% CI)	*n* (%)	aPR (95% CI)
**Any ICT tools**										
0	7 (87.5)	1	7 (87.5)	1	5 (62.5)	1	7 (87.5)	1	7 (87.5)	1
≥ 1	109 (55.1)	0.55 (0.10, 0.77) ***	97 (49.0)	0.47 (0.33, 0.66) ***	32 (16.2)	0.53 (0.43, 0.64)***	66 (33.3)	0.31 (0.19, 0.50)***	106 (53.5)	0.54 (0.40, 0.74) ***
**Text messages**										
No	36 (62.1)	1	34 (58.6)	1	15 (25.9)	1	24 (41.4)	1	36 (62.1)	1
Yes	80 (54.1)	0.82 (0.64, 1.07)	70 (47.3)	0.75 (0.57, 0.99) *	22 (14.9)	0.60 (0.34, 1.08)	49 (33.1)	0.80 (0.54, 1.18)	77 (52.0)	0.82 (0.64, 1.05)
**Voice calls**										
No	58 (63.7)	1	53 (58.2)	1	22 (24.2)	1	39 (42.9)	1	57 (62.6)	1
Yes	58 (50.4)	0.76 (0.59, 0.98) *	51 (44.4)	0.72 (0.55, 0.94) *	15 (13.0)	0.55 (0.29, 1.01)	34 (29.6)	0.69 (0.48, 0.99)*	56 (48.7)	0.76 (0.59, 0.98) *
**Video calls**										
No	61 (63.5)	1	54 (56.3)	1	26 (27.1)	1	43 (44.8)	1	62 (64.6)	1
Yes	55 (50.0)	0.72 (0.57, 0.92) **	50 (45.5)	0.73 (0.56, 0.96) **	11 (10.0)	0.36 (0.19, 0.69) **	30 (27.3)	0.57 (0.39, 0.83)**	51 (46.4)	0.67 (0.52, 0.86) **
**Picture messages**										
No	90 (60.8)	1	80 (54.1)	1	30 (20.3)	1	54 (36.5)	1	89 (60.1)	1
Yes	26 (44.8)	0.66 (0.48, 0.91) *	24 (41.4)	0.69 (0.49, 0.96) *	7 (12.1)	0.56 (0.26, 1.21)	19 (32.8)	0.87 (0.57, 1.31)	24 (41.4)	0.66 (0.47, 0.92) *
**Short videos**										
No	95 (58.6)	1	85 (52.5)	1	31 (19.1)	1	56 (34.6)	1	91 (56.2)	1
Yes	21 (47.7)	0.74 (0.53, 1.04)	19 (43.2)	0.70 (0.49, 1.02)	6 (13.6)	0.64 (0.28, 1.44)	17 (38.6)	0.89 (0.59, 1.36)	22 (50.0)	0.82 (0.59, 1.13)
**Emails**										
No	114 (56.2)	1	103 (50.7)	1	36 (17.7)	1	72 (35.5)	1	112 (55.2)	1
Yes	2 (66.7)	1.25 (0.57, 2.72)	1 (33.3)	0.63 (0.14, 2.76)	1 (33.3)	1.35 (0.50, 3.64)	1 (33.3)	1.01 (0.38, 2.72)	1 (33.3)	0.68 (0.14, 3.33)
**Number of ICT tools**	-	0.85 (0.77, 0.93) **	-	0.83 (0.75, 0.92) ***	-	0.67 (0.50, 0.89)**	-	0.84 (0.72, 0.97) *	-	0.84 (0.76, 0.92) ***

Missing data were excluded. * *P* < 0.05, ** *P* < 0.01, *** *P* < 0.001. aPR, adjusted prevalence ratio, adjusted for sex, age, education level, marital status, monthly household income, and household registration

The prevalence of mild symptoms and clinically significant conditions of all outcomes are shown in sTable (1) The results of the sensitivity analysis are shown in sTable (2) For the clinically significant detection of depression, anxiety, PTSD, and insomnia using higher cutoffs, most results remain similar but with stronger associations. Specifically, using any ICT tools was associated with even lower odds of depression, anxiety, PTSD, and insomnia (aPRs 0.17–0.41, all *P* ≤ 0.003). Both using video calls and more kinds of ICT tools were associated with even lower odds of depression, anxiety, and PTSD (video calls: aPRs 0.36–0.72, all *P* ≤ 0.02; more ICT tools: aPRs 0. 46 − 0.80, all *P* ≤ 0.02). Using voice calls was only associated with lower odds of PTSD (aPR 0.47, all *P* = 0.049).

## Discussion

This cross-sectional study was the first to examine how COVID-19 patients used ICT to communicate with family/friends and its associations with mental health and insomnia during their hospital quarantine. Different ICT use varied in sex, age, education levels, and monthly household income. Using any or more ICT tools to communicate with family/friends showed lower odds of depression, anxiety, PTSD, somatization symptoms, and insomnia, especially for using video calls. For early detection using costless prevention measures, this study used lower cutoffs to identify mild psychological symptoms and insomnia as the outcomes, and the robustness of findings were also verified using higher cutoff points for clinical diagnoses.

While previous studies only showed the potential benefits of ICT communication on personal happiness and family wellbeing in normal life [8, 37], this study extended its benefits on mental health and insomnia in the hospital setting. Quarantined patients in quarantine may experience more depression, anxiety, and less sleep due to uncertainties in their prognosis, concerns of long-term health effects, and social isolation from the outside world [38, 39]. ICT communication, such as exchanging messages, sharing photos or videos, or engaging in real-time conversations, helps foster the sense of belonging and connectedness by maintaining emotional ties with their family/friends [35, 36, 40]. Such connection can serve as a vital source of social support, a known protective social capital against mental problems [41, 42].

This study first highlighted the associations of using video calls with less PTSD, somatization symptoms, and insomnia in quarantined patients. Video calls can offer advantages in fostering social connections and emotional support [43, 44]. Most previous studies concentrated on how video calls affect the elder generation during the COVID-19 pandemic [40, 45], showing that video calls may play a more useful role than voice call in reducing mental health problems [46, 47]. Making video calls has been proven to be closer to face-to-face contact than other ICT tools. Based on Porges’s theory of social engagement and attachment, with the visual component, video calls can present crucial body languages like facial expressions and eye contact, so as to diminish perceived social distance [47].

This study adds evidence to the inverse care law in ICT use. Patients with lower education levels tended to have less ICT communication during their quarantine. While those with higher education levels may place a higher value on maintaining social connections and thus more actively use ICT tools for direct and convenient interaction, especially video calls [48]. This finding emphasizes the role of education in shaping COVID-19 patients’ communication choices. Educational backgrounds should be considered when implementing ICT related support to quarantined patients. Meanwhile, patients with lower monthly incomes are more likely to send/receive short videos. Such preference for short videos may stem from the desire for entertainment and distraction during isolation, as short videos can provide a quick and visually engaging form of content [49]. These findings have important implications for understanding ICT communication patterns and designing targeted interventions for quarantine people. By tailoring communication approaches to patients with specific demographics, we can optimize social engagement and maximize the benefits of technology-mediated communication during quarantine.

Consistent with the digital divide, older patients (≥ 55 years) tended to use less ICT tools during their quarantine. They may be less familiar or lack the knowledge of ICT communication and less convinced of the ICT benefits [50]. It is well known that the exacerbation of aging has evolved into a salient societal problem. Such a generation gap in ICT tools could be attributed to a lack of exposure to ICT or a preference for more traditional forms of communication in older patients. Previous studies have demonstrated that ICT self-efficacy can moderate the association between ICT usage and loneliness [51, 52]. Targeted interventions should be implemented to facilitate ICT adoption among elderly patients to maintain communication with family/friends, so as to decrease mental symptoms during quarantine.

Notably, during the COVID-19 pandemic, 96.3% of the quarantined patients used at least one type of ICT tools to communicate with family/friends, which was much higher than the general population in other areas, including 26.7% of Hong Kong Chinese adults using instant messages for family communication [37] and 18.9–21.2% of Japanese adults using remote communication [53]. Several reasons could account for the high ICT family communication. First, the survey was conducted in Shenzhen, a city with the highest internet and smartphone penetration rate (93.2% and 99.4%, respectively) in mainland China in 2020 [54], Second, most quarantined patients were aged below 55 years (83.4%) and were more proficient in ICT use than the older population. Third, before being transferred to the survey hospital, every 2–3 of these patients have been isolated in a ward for at least 2 weeks to better treat and prevent the spread of the COVID-19 disease. ICT was the only means of maintaining external communication during hospital quarantine. Under high social restrictions, additional support beyond remote ICT communication may be valuable for quarantined patients to alleviate mental distress.

This study has some limitations. First, the cross-sectional study design could not verify any causal relationship due to lack of chronological order. However, our findings were consistent with previous studies in normal life, showing the mental benefits of ICT communication for quarantined patients and providing evidence for possible non-pharmaceutical interventions using ICT to improve patients’ mental health during quarantine. Second, recall bias and selection bias were unavoidable for self-reported questionnaires. However, our findings add evidence to the inverse care law in ICT use [55], showing that older patients, or those with lower education or income levels used less ICT tools. Third, some results with no statistical significance may be due to the small sample size in this study. However, the associations of ICT use with mental health outcomes found in this study are consistent with previous studies [52, 56], showing that the directions of our findings were credible. Fourth, this study had a relatively low response rate, with only 29.8% of patients providing complete questionnaires and being included in the final analysis. Selection bias was possible as the included patients might have higher compliance and more attention to family communication and ICT use. While we had no access to the detailed information of the non-respondents, it is possible that they may differ from respondents in terms of personal characteristics, disease severity, or other characteristics. If such differences are substantial, they may limit the representativeness of the study sample and the generalizability of the findings. However, our findings showing that ICT use, especially using video calls, might benefit isolated patients’ mental health, are consistent with previous studies [52, 56]. Last, the generalizability of this study may be limited due to the differences in cultural context and quarantine policies. Evidence from Western settings is warranted to confirm our findings.

## Conclusion

This study shows that using any or more types of ICT to communicate with family/friends was associated with lower odds of depression, anxiety, PTSD, somatic symptoms, and insomnia for COVID-19 patients during hospital quarantine, especially using video calls. This study added evidence to the Inverse care law and digital divide in ICT use, showing that patients at older ages, lower education levels, and lower monthly incomes used less ICT communication and thus may need special attention during quarantine. Future studies in Western settings are warranted to confirm our findings.

## Electronic supplementary material

Below is the link to the electronic supplementary material.

Supplementary Material 1

## Data Availability

The datasets used and/or analysed during the current study are available from the corresponding author on reasonable request.

## Abbreviations

ICT: Information and communication technology
PTSD: Post-traumatic stress disorder
PHQ-9: The Patient Health Questionnaire
GAD-7: The Generalized Anxiety Disorder Scale
ISI: The Insomnia Severity Index
PCL-C: The PTSD Checklist-Civilian version
SSS-CN: The Somatic Symptom Scale-China
SD: Standard deviation
aPR: Adjusted prevalence ratio
CI: Confidence interval
