# Supplementary material for: ICT communication with family/friends and its associations with mental health and insomnia in COVID-19 patients during hospital quarantine: a cross-sectional study in Shenzhen, China
Source: BMC Psychiatry. 2025 Jul 1;25:648. doi: 10.1186/s12888-025-07079-6 (PMC12220049; doi:10.1186/s12888-025-07079-6)
Supplement: Supplementary file 1 — Supplementary Material 1 [file 12888_2025_7079_MOESM1_ESM.docx]

**sTable 1** The prevalence of mental health problems and insomnia using different cutoffs

| **Mental health problems and insomnia** | **n (%) using lower cutoffs for mild symptoms^a^** | **n (%) using higher cutoffs for clinically significant conditions** |
| --- | --- | --- |
| **Depression** |  |  |
| No | 90 (43.7) | 161 (74.2) |
| Yes | 116 (56.3) | 56 (25.8) |
| **Anxiety** |  |  |
| No | 102 (49.5) | 167 (81.5) |
| Yes | 104 (50.5) | 38 (18.5) |
| **Post-traumatic syndrome** |  |  |
| No | 169 (82.0) | 188 (86.6) |
| Yes | 37 (18.0) | 29 (13.4) |
| **Somatization symptoms^** |  |  |
| No | 133 (64.6) | - |
| Yes | 73 (35.4) | - |
| **Insomnia** |  |  |
| No | 93 (45.2) | 144 (66.4) |
| Yes | 113 (44.9) | 73 (33.6) |

Depression, anxiety, post-traumatic syndrome, and insomnia were considered as having mild symptoms using the lower cutoffs of ≥5, ≥5, ≥38, and ≥8 for the PHQ-9, GAQ-7, PCL-C, and ISI scores, respectively, and as having clinically significant conditions using the higher cutoffs of ≥10, ≥10, ≥44, and ≥11.

**^** Somatization symptoms remained using the same cutoffs of ≥30 for the SSS-CN scores.

**sTable 2** Using various ICT tools to contact family/friends links to mental health, insomnia, somatization. (N=206)

| Different ICT tools | Depression | | Anxiety | | Post-traumatic syndrome | | Insomnia | |
| --- | --- | --- | --- | --- | --- | --- | --- | --- |
|  | n (%) | aPR (95% CI) | n (%) | aPR (95% CI) | n (%) | aPR (95% CI) | n (%) | aPR (95% CI) |
| **Any ICT tools** |  |  |  |  |  |  |  |  |
| 0 | 5 (62.5) | 1 | 5 (62.5) | 1 | 5 (62.5) | 1 | 5 (62.5) | 1 |
| ≥1 | 51 (24.4) | 0.36 (0.20, 0.64)** | 33 (16.8) | 0.25 (0.13, 0.48)*** | 24 (11.5) | 0.17 (0.08, 0.37)*** | 68 (32.5) | 0.41 (0.23, 0.73)** |
| **Text messages** |  |  |  |  |  |  |  |  |
| No | 15 (25.4) | 1 | 12 (21.8) | 1 | 14 (23.7) | 1 | 20 (33.9) | 1 |
| Yes | 41 (26.0) | 0.99 (0.59, 1.65) | 26 (17.3) | 0.81 (0.45, 1.48)* | 15 (9.5) | 0.38 (0.2, 0.74)** | 53 (33.5) | 0.95 (0.63, 1.43) |
| **Voice calls** |  |  |  |  |  |  |  |  |
| No | 30 (32.3) | 1 | 21 (23.9) | 1 | 18 (19.4) | 1 | 36 (38.7) | 1 |
| Yes | 26 (21.0) | 0.66 (0.42, 1.04) | 17 (14.5) | 0.68 (0.39, 1.17) | 11 (8.9) | 0.47 (0.22, 1.00)* | 37 (29.8) | 0.74 (0.51, 1.07) |
| **Video calls** |  |  |  |  |  |  |  |  |
| No | 36 (35.6) | 1 | 25 (25.8) | 1 | 20 (19.8) | 1 | 38 (37.6) | 1 |
| Yes | 20 (17.2) | 0.43 (0.27, 0.69)*** | 13 (12.0) | 0.42 (0.24, 0.74)** | 9 (7.8) | 0.36 (0.17, 0.77)** | 35 (30.2) | 0.72 (0.49, 1.04) |
| **Picture messages** |  |  |  |  |  |  |  |  |
| No | 41 (26.8) | 1 | 28 (19.6) | 1 | 22 (14.4) | 1 | 55 (36.0) | 1 |
| Yes | 15 (23.4) | 0.78 (0.46, 1.32) | 10 (16.1) | 0.74 (0.39, 1.42) | 7 (10.9) | 0.69 (0.3, 1.58) | 18 (28.1) | 0.72 (0.46, 1.13) |
| **Short videos** |  |  |  |  |  |  |  |  |
| No | 45 (26.8) | 1 | 30 (19.1) | 1 | 23 (13.7) | 1 | 56 (33.3) | 1 |
| Yes | 11 (22.5) | 0.74 (0.42, 1.30) | 8 (16.7) | 0.75 (0.38, 1.51) | 6 (12.2) | 0.83 (0.36, 1.93) | 17 (34.7) | 0.89 (0.56, 1.39) |
| **Emails** |  |  |  |  |  |  |  |  |
| No | 56 (26.2) | - | 38 (18.8) | - | 29 (13.6) | - | 73 (34.1) | - |
| Yes | 0 (0) | -^ | 0 (0) | -^ | 0 (0) | -^ | 0 (0) | -^ |
| **Number of ICT tools** | - | 0.80 (0.67, 0.96)* | - | 0.79 (0.63, 0.98)* | - | 0.64 (0.46, 0.91)** | - | 0.87 (0.76, 1.00) |

Missing data were excluded. * *P*<0.05, ** *P*<0.01, *** *P*<0.001. ^The aPRs were lower than 0.01 because patients using emails were quite scarce and no positive cases for these outcomes.

aPR, adjusted prevalence ratio, adjusted for sex, age, education level, marital status, monthly household income, and household registration.
